# Association between the stress hyperglycemia ratio and severity of coronary artery disease under different glucose metabolic states

**DOI:** 10.1186/s12933-023-01759-x

**Published:** 2023-02-08

**Authors:** Yu Zhang, Haiyan Song, Jing Bai, Jiahui Xiu, Ganggang Wu, Liao Zhang, Yunhe Wu, Ying Qu

**Affiliations:** Department of Endocrinology, The Second Affiliated Hospital of Harbin Medical University, Harbin, China

**Keywords:** Stress hyperglycemia ratio, Coronary artery disease, Normal glucose regulation, Pre-diabetes mellitus, Diabetes mellitus

## Abstract

**Background:**

Stress hyperglycemia ratio (SHR) is significantly related to adverse cardiovascular clinical outcomes and increased in-hospital mortality. However, the relationship between SHR and coronary artery disease (CAD) severity has hitherto not been reported. This study sought to clarify the relationship between the SHR and CAD severity of individuals with different glucose metabolic statuses.

**Methods:**

A retrospective analysis was performed on 987 patients who underwent coronary angiography (CAG) from October 2020 to May 2022. Based on CAG results, patients were divided into single-vessel CAD and multi-vessel CAD groups. All subjects were stratified into three groups according to the tertiles of the SHR (T1 group: SHR < 0.930; T2 group: 0.930 ≤ SHR < 1.154; T3 group: 1.154 ≤ SHR). Moreover, according to glucose metabolism status, study subjects were divided into normal glucose regulation (NGR), pre-diabetes mellitus (pre-DM) and diabetes mellitus (DM) groups. Finally, the correlation between SHR and CAD severity was analyzed by logistic regression analysis and receiver operating characteristic (ROC) curve.

**Results:**

The results showed significantly higher SHR in the multi-vessel CAD group than in the single-vessel group. Logistic regression analysis showed that SHR was an independent risk factor for multi-vessel CAD when used as a continuous variable (OR, 4.047; 95% CI 2.137–7.663; P < 0.001). After adjusting for risk factors, the risk of multi-vessel CAD in the T2 and T3 groups was 1.939-fold (95% CI 1.341–2.804; P < 0.001) and 1.860-fold (95% CI 1.272–2.719; P = 0.001) higher than in the T1 group, respectively. The area under the curve (AUC) of ROC plots was 0.613 for SHR. In addition, SHR was significantly correlated with an increased risk of multi-vessel CAD in the pre-DM and DM groups.

**Conclusions:**

Our study indicated that SHR was significantly correlated with the risk of multi-vessel CAD and predicted CAD severity, especially in pre-DM and DM patients.

## Introduction

The poor dietary habits and lifestyle of the general population, and global population aging account for the significant increase in morbidity and mortality attributed to coronary artery disease (CAD) in recent years [1], bringing a serious economic and health burden on our public health systems. Indeed, it is well-established that coronary heart disease (CHD) patients with abnormal glucose metabolism have a higher risk of cardiovascular adverse events [2, 3]. It is widely acknowledged that the number of stenotic vessels determines the severity and prognosis of CAD [3, 4]. Therefore, it is very important for identify high-risk populations with multi-vessel CAD, especially those with abnormal glucose metabolism, to more accurately and efficiently judge the condition of patients and provide optimal treatment.

Stress hyperglycemia represents a transient physiologic response to acute diseases [5]. There is a growing consensus that stress hyperglycemia is associated with adverse cardiovascular clinical outcomes and increased in-hospital mortality [5–10]. Chronic hyperglycemia in diabetic patients is a well-defined risk factor for adverse cardiovascular events [11], but those with stress hyperglycemia are at higher risk of adverse outcomes than those with pre-existing diabetes [5, 6]. Stress hyperglycemia is greatly affected by the glycemia background. To better evaluate the actual blood glucose status of patients, researchers have proposed to use the stress hyperglycemia ratio (SHR) to estimate relative hyperglycemia in patients with or without diabetes to identify and quantify stress hyperglycemia [12]. Current evidence suggests that the glycemia background does not affect the relationship between SHR and critical illness. Studies have also confirmed that relative hyperglycemia measured by SHR was more associated with critical illness than absolute hyperglycemia and was a better biomarker for critical illness than absolute hyperglycemia [12]. Moreover, overwhelming evidence substantiates that SHR is significantly related to adverse cardiovascular outcomes and related mortality [11, 13–15].

However, the relationship between SHR and CAD severity remains largely unclear, warranting further research. This study sought to clarify the correlation between SHR and CAD severity and the strength of the correlation under different glucose metabolic conditions.

## Methods

### Study design and population

**Fig. 1:**
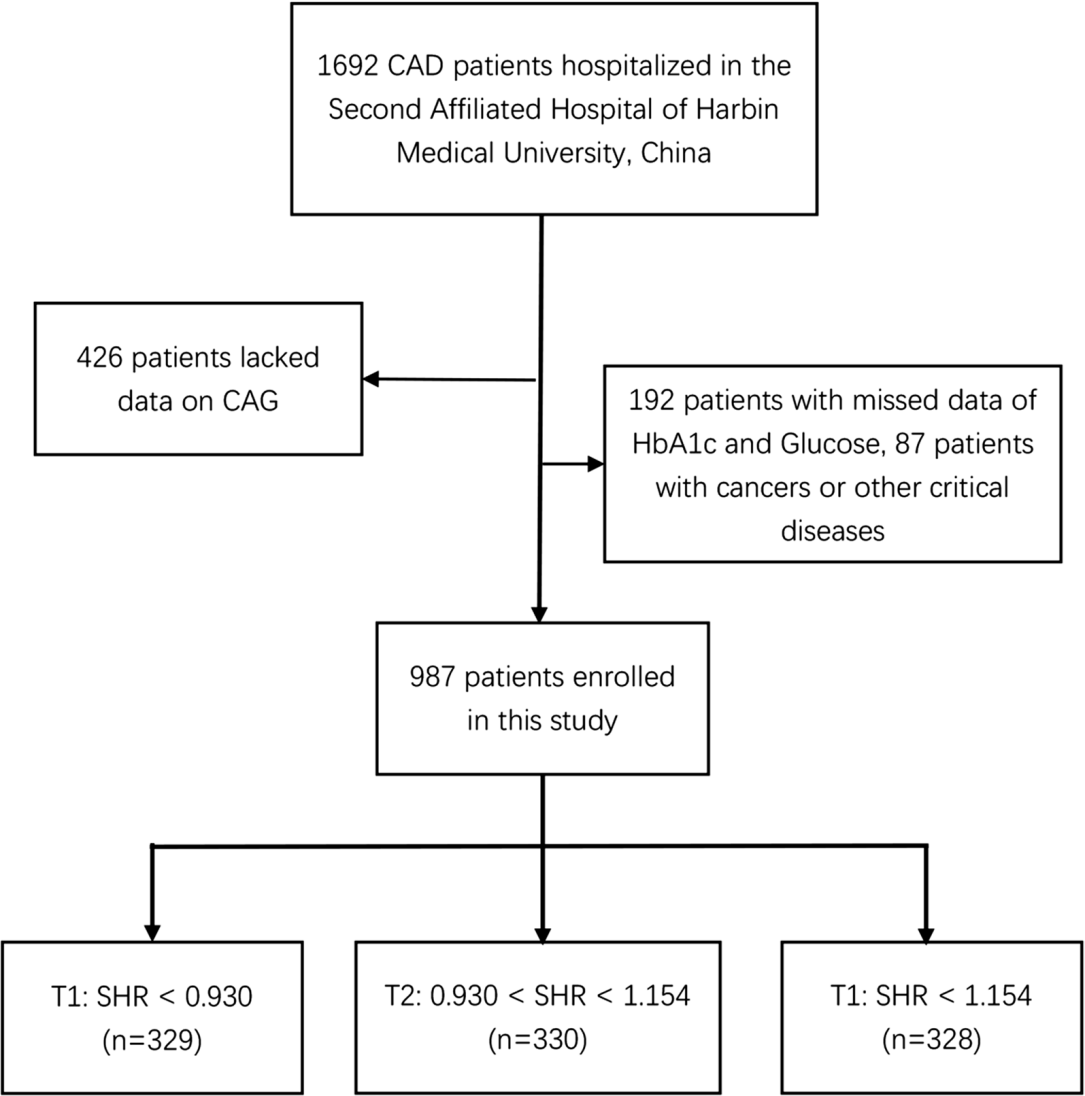
Flow chart of patient recruitment. *CAD* Coronary artery disease, *CAG* coronary angiography, *HbA1c* glycated hemoglobin A1c, *SHR* stress hyperglycemia ratio

This retrospective study conformed to the Declaration of Helsinki and was approved by the Ethics Committee in the Second Affiliated Hospital of Harbin Medical University. 1692 CAD patients hospitalized in the Second Affiliated Hospital of Harbin Medical University from October 2020 to May 2022 were selected. The exclusion criteria were as follows: (1) patients under 18 years of age; (2) patients with missing data on glycated hemoglobin A1c (HbA1c), admission glucose and coronary angiography (CAG); (3) patients with life-threatening diseases such as tumors, infectious diseases or severe liver or kidney diseases. Ultimately, 987 eligible participants were enrolled in the study. Study subjects were grouped based on tertiles as follows: T1 group, SHR < 0.930 (n = 329); T2 group, SHR ≥ 0.930 to < 1.154 (n = 330); and T3 group, SHR ≥ 1.154 (n = 328). The study flow chart is shown in Fig. 1.

### Data collection and definitions

Sociodemographic characteristics (age, sex, height, weight, smoking status, and alcohol consumption), medical history (hypertension, diabetes, cancer and other previous medical conditions), and laboratory results were derived from hospital medical records. Admission glucose was measured for the first time within 24 h after admission. Moreover, we recorded the patients' clinical data, including CAG results, blood-related indicators and echocardiography-related parameters. Blood-related indicators included admission glucose, HbA1c, C-reactive protein (CRP), B-type natriuretic peptide (BNP), creatinine (Cr), uric acid (UA), total cholesterol (TC), triglycerides (TG), high-density lipoprotein cholesterol (HDL-C), and low-density lipoprotein cholesterol (LDL-C). Echocardiographic data included left atrial diameter (LAD), left ventricular end-diastolic diameter (LVDd), left ventricular systolic diameter (LVDs), interventricular septal thickness (IVS), left posterior wall thickness (LVPW), and left ventricular ejection fraction (LVEF) [12].

$${\text{SHR}}\, = \,{\text{admission glucose }}\left( {{\text{mmol}}/{\text{L}}} \right)/[{1}.{59}\, \times \,{\text{HbA1c }}\left( \% \right){-\!\!-}{2}.{59}]$$

$${\text{Body mass index }}\left( {{\text{BMI}}} \right)\, = \,{\text{weight }}\left( {{\text{kg}}} \right)/{\text{height}}^{{2}} ({\text{m}}^{{2}} )$$

According to glucose metabolism status, study subjects were divided into normal glucose regulation (NGR), pre-diabetes mellitus (pre-DM) and diabetes mellitus (DM) groups. NGR was defined as HbA1c < 5.7% and no previous history of diabetes; 5.7% ≤ HbA1c < 6.5% and without a history of diabetes were classified as pre-DM; patients with a history of diabetes or HbA1c ≥ 6.5% were classified as DM [16].

It is well-established that the three main coronary arteries include the left anterior descending, left circumflex, and right coronary arteries, and ≥ 50% stenosis in at least one main coronary artery is considered CAD. In our study, based on the CAG results, patients with one lesion were defined as single-vessel CAD, and the involvement of two or more coronary arteries was defined as multi-vessel CAD. However, multi-vessel CAD was also defined with left main coronary artery stenosis ≥ 50% [3].

### Statistical analysis

Continuous variables are expressed as mean ± standard deviation (SD) or median and interquartile range (IQR). Categorical variables are expressed as numbers and percentages. χ^2^ test was used for comparing categorical variables, and the* t*-test, analysis of variance, Mann–Whitney U or Kruskal–Wallis H test were used for continuous variable. To analyze the association between SHR and CAD severity, odds ratios (OR) and 95% confidence intervals (CI) were calculated using logistic regression. The receiver operating characteristic (ROC) and the area under the curve (AUC) were used to determine the sensitivity and specificity of SHR in predicting CAD severity. All data were statistically analyzed by SPSS 26.0 (IBM Corp, New York, NY, USA). A P-value < 0.05 was statistically significant.

## Results

### Baseline characteristics according to single-vessel or multi-vessel CAD

A total of 987 patients were included in this study, with a median age of 62 (IQR, 58–68 years), exhibiting male predominance (71.5%). Based on the CAG results, single- and multi-vessel lesions were found in 27.36% and 72.64% of patients, respectively. Based on the glucose metabolism status, the patients were divided into the NGR group (26.6%), the pre-DM group (34%), and the DM group (39.4%). Table 1 shows the baseline characteristics of the single-vessel and multi-vessel CAD groups. Compared with the single-vessel CAD group, patients in the multi-vessel CAD group were significantly older, had a higher prevalence of hypertension, and were more prone to glucose metabolism disorders (P < 0.05). In addition, patients in the multi-vessel CAD group had significantly higher HbA1c, glucose, BNP, D-dimer, LAD and SHR, and lower LVEF than their counterparts in the single-vessel group (P < 0.05) (Table 1).

**Table 1:** Baseline characteristics according to single-vessel or multi-vessel CAD

	Total (n = 987)	Single-vessel CAD (n = 270)	Multi-vessel CAD (n = 717)	P-value
Age (years)	62 (54, 69)	58 (51, 67)	63 (56, 70)	< 0.001
Male (n, %)	706 (71.50%)	205 (75.90%)	501 (69.90%)	0.060
NGR (n, %)	262 (26.60%)	85 (31.50%)	177 (24.70%)	< 0.001
Pre-DM (n, %)	336 (34%)	114 (42.20%)	222 (31%)	
DM (n, %)	389 (39.40%)	71 (26.30%)	318 (44.40%)	
Smoking (n, %)	428 (43.40%)	130 (48.10%)	298 (41.60%)	0.063
Drinking (n, %)	134 (13.60%)	41 (15.20%)	93 (13%)	0.365
Hypertension (n, %)	475 (48.10%)	114 (42.20%)	361 (50.30%)	0.023
BMI (kg/m^2^)	25.39 (23.03, 28.01)	25.10 (23.12, 28.08)	25.39 (23.03, 27.97)	0.762
HbA1c (%)	6 (5.60, 7)	5.80 (5.53, 6.30)	6.10 (5.60, 7.30)	< 0.001
Glucose (mmol/l)	7.33 (5.99, 9.85)	6.62 (5.48, 7.97)	7.67 (6.21, 10.69)	< 0.001
TC (mmol/l)	4.71 (3.95, 5.51)	4.75 (3.99, 5.42)	4.68 (3.93, 5.52)	0.924
TG (mmol/l)	1.29 (0.91, 1.81)	1.28 (0.96, 1.77)	1.30 (0.90, 1.85)	0.959
HDL-C(mmol/l)	1.10 (0.92, 1.28)	1.14 (0.94, 1.33)	1.09 (0.91, 1.26)	0.074
LDL-C(mmol/l)	3.07 (2.44, 3.73)	3.10 (2.48, 3.66)	3.05 (2.39, 3.77)	0.957
UA (umol/l)	376.05 (307.08, 459.03)	376 (313.45, 449.48)	376.20 (304.95, 461.43)	0.782
Cr (umol/l)	80 (67, 97.25)	78 (66, 90)	81 (67, 100)	0.091
BNP (pg/ml)	732 (227.50, 2240)	488 (175, 1477.25)	867 (243, 2544.50)	< 0.001
D-dimer (ug/ml)	106 (58, 224.25)	91.50 (50, 212.75)	110.50 (61, 225.25)	0.029
CRP (mg/l)	6.46 (2.45, 13.48)	5.72 (2.05, 13.07)	6.79 (2.64, 13.70)	0.086
LAD (mm)	35.80 (32.58, 38.70)	35.25 (32.13, 38.28)	36 (32.80, 38.90)	0.030
LVDd (mm)	46.90 (43.90, 50.20)	47 (44, 49.70)	46.85 (43.90, 50.50)	0.480
LVDs (mm)	30 (26.10, 34.50)	30 (25.73, 33.95)	30 (26.20, 34.63)	0.103
IVS (mm)	10.80 (10.10, 11.70)	10.70 (10, 11.60)	10.90 (10.10, 11.70)	0.128
LVPW (mm)	10.50 (10, 11.20)	10.50 (10, 11.08)	10.50 (10, 11.20)	0.624
LVEF (%)	58 (58.88, 62)	58.40 (51.63, 62)	58 (48, 62)	0.028
SHR	1.05 (0.88, 1.23)	0.99 (0.81, 1.17)	1.07 (0.90, 1.27)	< 0.001

*CAD* Coronary artery disease, *NGR* normal glucose regulation, *Pre-DM* pre-diabetes mellitus, *DM* diabetes mellitus, *BMI* body mass index, *HbA1c* glycated hemoglobin A1c, *TC* total cholesterol, *TG* triglycerides, *HDL-C* high-density lipoprotein cholesterol, *LDL-C* low-density lipoprotein cholesterol, *UA* uric acid, *Cr* creatinine, *BNP* B-type natriuretic peptide, *CRP* C-reactive protein, *LAD* left atrial diameter, *LVDd* left ventricular end diastolic diameter, *LVDs* left ventricular systolic diameter, *IVS* interventricular septal thickness, *LVPW* left posterior wall thickness, *LVEF* left ventricular ejection fraction, *SHR* stress hyperglycemia ratio

### Baseline characteristics according to the tertiles of SHR

Table 2 shows the baseline characteristics according to the tertiles of SHR. A significantly great number of males and patients with glucose metabolism disorders were found in the T3 group (P < 0.05). The levels of HbA1c, glucose, TC, HDL-C and LDL-C in the T3 group were significantly higher than in the T1 group (P < 0.05), and the proportion of multi-vessel CAD cases was higher in the T3 group than in the other groups (P < 0.05).

**Table 2:** Baseline characteristics according to the tertiles of SHR

	Total (n = 987)	T1(n = 329)	T2(n = 330)	T3(n = 328)	P-value
Age (years)	62 (54, 69)	63 (55, 69)	62(53, 69.25)	63 (55, 69)	0.465
Male (n, %)	706 (71.50%)	250 (76%)	242(73.30%)	214 (65.20%)	0.006
NGR (n, %)	262 (26.60%)	79 (24%)	112(33.9)	71 (21.60%)	< 0.001
Pre-DM (n, %)	336 (34%)	132 (40.10%)	117 (35.50%)	87 (26.50%)	
DM (n, %)	389 (39.40%)	118 (35.90%)	101 (30.60%)	170 (51.80%)	
Smoking (n, %)	428 (43.40%)	168 (51.10%)	147 (44.50%)	113 (34.50%)	< 0.001
Drinking (n, %)	134 (13.60%)	48 (14.60%)	46 (13.9%)	40 (12.20%)	0.651
Hypertension (n, %)	475 (48.10%)	162 (49.20%)	154 (46.70%)	159 (48.50%)	0.794
BMI (kg/m^2^)	25.39 (23.03, 28.01)	25.09 (23.12, 27.78)	25.75 (23.03, 28.41)	25.39 (22.76, 27.55)	0.544
HbA1c (%)	6 (5.60, 7)	5.90 (5.60, 6.70)	5.80 (5.50, 6.53)	6.20 (5.70, 7.60)	< 0.001
Glucose (mmol/l)	7.33 (5.99, 9.85)	5.74 (5.19, 6.49)	7.05 (6.39, 8.34)	10.22 (8.28, 13.82)	< 0.001
TC (mmol/l)	4.71 (3.95, 5.51)	4.47 (3.81, 5.28)	4.84 (4.07, 5.68)	4.83 (4.01, 5.52)	0.002
TG (mmol/l)	1.29 (0.91, 1.81)	1.41 (1.04, 1.99)	1.21 (0.88, 1.68)	1.23 (0.86, 1.72)	< 0.001
HDL-C(mmol/l)	1.10 (0.92, 1.28)	1 (0.86, 1.22)	1.16(0.97, 1.30)	1.13 (0.96, 1.29)	< 0.001
LDL-C(mmol/l)	3.07 (2.44, 3.73)	2.87 (2.22, 3.52)	3.20 (2.58, 3.90)	3.21 (2.51, 3.72)	< 0.001
UA (umol/l)	376.05 (307.08, 459.03)	371.40 (306.75, 462.60)	384.05 (306.58, 463.85)	371.80 (307.70, 448.10)	0.694
Cr (umol/l)	80 (67, 97.25)	79 (66.50, 97.50)	81 (66, 97)	82 (68, 99)	0.446
BNP (pg/ml)	732 (227.50, 2240)	633 (217, 1831.50)	744 (242, 2007.25)	904 (217, 2808)	0.131
D-dimer (ug/ml)	106 (58, 224.25)	105 (54.50, 202.50)	109 (59.75, 217)	101 (58, 241)	0.945
CRP (mg/l)	6.46 (2.45, 13.48)	6.79 (2.35, 13.28)	6.49 (2.12, 13.61)	6.30 (2.73, 13.70)	0.951
LAD (mm)	35.80 (32.58, 38.70)	35.60 (32.10, 38.60)	35.85 (32.65, 38.80)	36 (33, 38.70)	0.415
LVDd (mm)	46.90 (43.90, 50.20)	46.90 (43.80, 50.15)	47 (44, 50.20)	46.60 (44, 50.50)	0.938
LVDs (mm)	30 (26.10, 34.50)	29.70 (25.90, 34.75)	30 (26.18, 34.75)	30.20 (26.30, 34.90)	0.868
IVS (mm)	10.80 (10.10, 11.70)	10.80 (10.10, 11.60)	10.80 (10.10, 11.70)	10.80 (10.10, 11.80)	0.984
LVPW (mm)	10.50 (10, 11.20)	10.50 (10, 11.05)	10.50(10, 11.20)	10.50 (10, 11.20)	0.945
LVEF (%)	58 (58.88, 62)	58.50 (52, 62)	58 (49, 61)	57 (47, 62)	0.069
Multi-vessel CAD (n, %)	717 (72.60%)	210 (29.30%)	249 (34.70%)	258 (36%)	< 0.001

*SHR* stress hyperglycemia ratio, *NGR* normal glucose regulation, *Pre-DM* pre-diabetes mellitus, *DM* diabetes mellitus, *BMI* body mass index, HbA1c, glycated hemoglobin A1c, *TC* total cholesterol, *TG* triglycerides, *HDL-C* high-density lipoprotein cholesterol, *LDL-C* low-density lipoprotein cholesterol, *UA* uric acid, *Cr* creatinine, *BNP* B-type natriuretic peptide, *CRP* C-reactive protein, *LAD* left atrial diameter, *LVDd* left ventricular end diastolic diameter, *LVDs* left ventricular systolic diameter, *IVS* interventricular septal thickness, *LVPW* left posterior wall thickness, *LVEF* left ventricular ejection fraction, *CAD* Coronary artery disease

### Relationship between CAD severity and various risk factors

The association between CAD severity and various risk factors was analyzed by univariate logistic regression, taking the multi-vessel CAD as the dependent variable. We found that age, glucose metabolism state, hypertension, HbA1c, BNP, LAD and SHR were positively correlated with multi-vessel CAD (P < 0.05), and HDL-C was negatively correlated with multi-vessel CAD (P < 0.05) (Table 3).

**Table 3:** Relationship between CAD severity and various risk factors

Variables	Multi-vessel coronary artery disease		
	OR(95%CI)	β	P-value
Age	1.033 (1.020–1.046)	0.032	< 0.001
Sex			
Male	Reference		
Female	1.360 (0.986–1.875)	0.307	0.061
Glucose metabolism state			
NGR	Reference		
Pre-DM	0.935 (0.663–1.318)	− 0.067	0.702
DM	2.151 (1.493–3.098)	0.766	< 0.001
Smoking			
NO	Reference		
YES	0.766 (0.578–1.015)	− 0.267	0.063
Drinking			
NO	Reference		
YES	0.832 (0.559–1.239)	− 0.183	0.366
Hypertension			
NO	Reference		
YES	1.388 (1.046–1.840)	0.328	0.023
BMI	1.015 (0.971–1.061)	0.015	0.502
HbA1c	1.293 (1.148–1.456)	0.257	< 0.001
Glucose	1.180 (1.119–1.245)	0.166	< 0.001
TC	1.005 (0.897–1.126)	0.005	0.936
TG	1.009 (0.884–1.151)	0.009	0.896
HDL-C	0.568 (0.344–0.938)	− 0.566	0.027
LDL-C	0.996 (0.868–1.144)	− 0.004	0.960
UA	1.002 (0.999–1.006)	0.002	0.117
Cr	0.999 (0.998–1.001)	< − 0.001	0.972
BNP	1.000 (1.000–1.001)	< 0.001	0.009
D-dimer	1.000 (0.999–1.001)	< 0.001	0.763
CRP	1.020 (0.994–1.047)	0.020	0.135
LAD	1.032 (1.000–1.064)	0.031	0.048
LVDd	1.013 (0.988–1.039)	0.013	0.319
LVDs	1.021 (0.999–1.043)	0.021	0.053
IVS	1.068 (0.962–1.187)	0.066	0.216
LVPW	1.021 (0.909–1.146)	0.020	0.731
LVEF	0.999 (0.999–1.000)	< − 0.001	0.053
SHR	4.590 (2.598–8.111)	1.524	< 0.001

*OR* odds ratios, *CI* confidence interval, *β* regression coefficient, *NGR* normal glucose regulation, *Pre-DM* pre-diabetes mellitus, *DM* diabetes mellitus, *HbA1c* glycated hemoglobin A1c, *BMI* body mass index, *TC* total cholesterol, *TG* triglycerides, *HDL-C* high-density lipoprotein cholesterol, *LDL-C* low-density lipoprotein cholesterol, *UA* uric acid, *Cr* creatinine, *LAD* left atrial diameter, *LVDd* left ventricular end diastolic diameter, *LVDs* left ventricular systolic diameter, *IVS* interventricular septal thickness, *LVPW* left posterior wall thickness, *LVEF* left ventricular ejection fraction, *BNP* B-type natriuretic peptide, *CRP* C-reactive protein, *SHR* stress hyperglycemia ratio

### Association between SHR and CAD severity

As shown in Table 4, multivariate logistic regression analysis was constructed to analyze the correlation between SHR and CAD severity. Multi-vessel CAD was used as the dependent variable, and there was no multicollinearity between the independent variables. Confounding factors were not adjusted in model 1, age and sex were adjusted for model 2, and model 3 was adjusted for age, sex, glucose metabolism state, hypertension, smoking, HDL-C, BNP, LAD, LVDs, and LVEF. We found that SHR was an independent risk factor for multi-vessel CAD when used as a continuous variable (P < 0.05). When SHR was a categorical variable, the risk of multi-vessel CAD in the T2 and T3 groups was higher than T1. After adjusting for all relevant risk factors in model 3, the risk of multi-vessel CAD in the T2 and T3 groups was 1.939 (95% CI 1.341–2.804; P < 0.001) and 1.860 (95% CI 1.272–2.719; P = 0.001) fold higher than in the T1 group, respectively (Table 4).

**Table 4:** Association between SHR and CAD severity

Variables	Multi-vessel coronary artery disease					
	Model 1		Model 2		Model 3	
	OR (95% CI)	P-value	OR (95% CI)	P-value	OR (95% CI)	P-value
SHR	4.590 (2.598–8.111)	< 0.001	4.567 (2.555–8.163)	< 0.001	4.047 (2.137–7.663)	< 0.001
T1	Reference		Reference		Reference	
T2	1.742 (1.244–2.440)	0.001	1.813 (1.287–2.554)	0.001	1.939 (1.341–2.804)	< 0.001
T3	2.089 (1.476–2.955)	< 0.001	2.061 (1.449–2.932)	< 0.001	1.860 (1.272–2.719)	0.001

*OR* odds ratios, *CI* confidence interval, *SHR* stress hyperglycemia ratio

Model 1: unadjusted;

Model 2: adjusted for age and sex;

Model 3: adjusted for age, sex, glucose metabolism state, hypertension, smoking, HDL-C, BNP, LAD, LVDs, LVEF

T1: SHR < 0.930; T2: 0.930 ≤ SHR < 1.154; T3: 1.154 ≤ SHR

### The predictive value of SHR for CAD severity

**Fig. 2:**
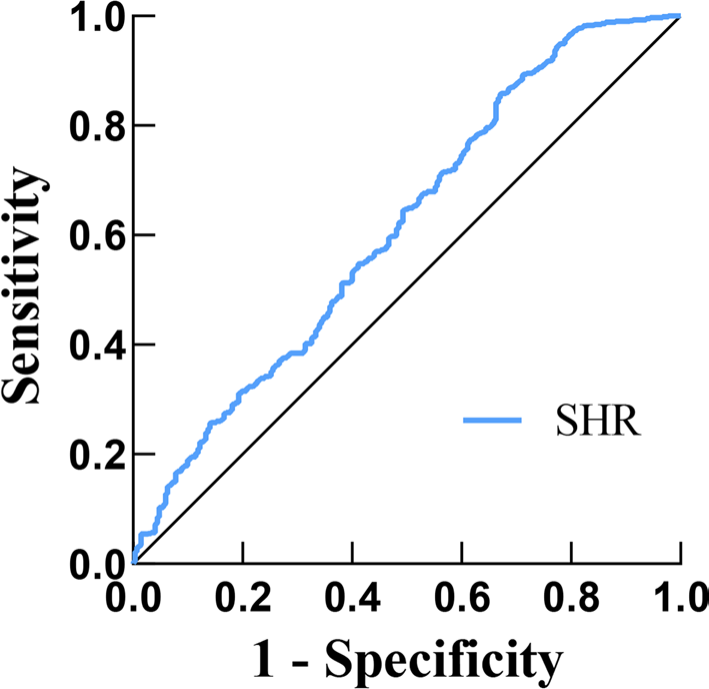
ROC curve for the use of SHR in the detection of multi-vessel CAD

The ROC curve for multi-vessel CAD and SHR is shown in Fig. 2. The AUC of multi-vessel CAD evaluated by SHR was 0.613 (95% CI 0.572–0.653), yielding an optimal cut-off value of 0.840 at a sensitivity of 85.8% and a specificity of 33% (P < 0.05) (Table 5).

**Table 5:** The predictive value of SHR for CAD severity

Variable	AUC	95% CI	Cut-off value	Sensitivity (%)	Specificity (%)
SHR	0.613	0.572–0.653	0.840	85.8	33.0

*AUC* area under the curve, *CI* confidence interval, *SHR* stress hyperglycemia ratio

### Correlations between SHR and CAD severity in different glucose metabolism states

These patients were divided into NGR, pre-DM and DM groups according to their glucose metabolic status. Logistic regression analysis was performed on the three groups with multi-vessel CAD as the dependent variable. In the NGR group, no significant association was found between SHR and the occurrence of multi-vessel CAD. In the pre-DM group, it was observed that SHR was an independent risk factor for multi-vessel CAD as a continuous variable (P < 0.05). And as a categorical variable, both T2 and T3 groups were correlated with an increased risk of multi-vessel CAD than the T1 group (P < 0.05). In the DM group, we also found that SHR was statistically significantly correlated with the increased risk of multi-vessel CAD (P < 0.05). Compared with the T1 group, T2 and T3 groups were more significantly correlated with the occurrence of multi-vessel CAD in models 4 and 5. However, after adjusting for all confounding risk factors, only the T3 group was associated with a significantly increased risk of multi-vessel CAD than the T1 group in model 6 (P < 0.05) (Table 6).

**Table 6:** Correlations between SHR and CAD severity in different glucose metabolism states

Glucose regulation state	Variables	Multi-vessel coronary artery disease					
		Model 4		Model 5		Model 6	
		OR (95% CI)	P-value	OR (95% CI)	P-value	OR (95% CI)	P-value
NGR	SHR	2.982 (0.911–9.759)	0.071	2.903 (0.845–9.982)	0.091	3.715 (0.957–14.412)	0.058
	T1	Reference		Reference		Reference	
	T2	1.546 (0.842–2.838)	0.16	1.534 (0.809–2.910)	0.190	1.879 (0.944–3.740)	0.073
	T3	1.538 (0.778–3.038)	0.216	1.526 (0.750–3.106)	0.244	1.828 (0.854–3.914)	0.120
Pre-DM	SHR	5.655 (1.899–16.837)	0.002	5.536 (1.838–16.674)	0.002	5.833 (1.805–18.852)	0.003
	T1	Reference		Reference		Reference	
	T2	1.799 (1.061–3.049)	0.029	1.876 (1.092–3.223)	0.023	1.905 (1.077–3.370)	0.027
	T3	1.934 (1.079–3.466)	0.027	1.871 (1.034–3.386)	0.038	1.890 (1.012–3.528)	0.046
DM	SHR	3.623 (1.527–8.595)	0.003	3.722 (1.561–8.874)	0.003	3.855 (1.494–9.947)	0.005
	T1	Reference		Reference		Reference	
	T2	2.312 (1.154–4.634)	0.018	2.309 (1.150–4.637)	0.019	1.983 (0.935–4.201)	0.074
	T3	2.158 (1.200–3.883)	0.010	2.190 (1.214–3.951)	0.009	2.059 (1.063–3.988)	0.032

*OR* odds ratios, *CI* confidence interval, *SHR* stress hyperglycemia ratio, *NGR* normal glucose regulation, *Pre-DM* pre-diabetes mellitus, *DM* diabetes mellitus

Model 4: unadjusted;

Model 5: adjusted for age and sex;

Model 6: adjusted for age, sex, hypertension, smoking, HDL-C, BNP, LAD, LVDs, LVEF

## Discussion

The present study showed that SHR was significantly associated with the severity of CAD, especially in the pre-DM and DM groups. Importantly, this is the first study to reveal the relationship between SHR and the risk of multi-vessel CAD to our best knowledge.

Herein, we observed that patients in the multi-vessel CAD group were older, had higher SHR, and were more prone to glucose metabolism disorders than the single-vessel group. Multivariate logistic regression analysis revealed that SHR was significantly associated with the risk of multi-vessel CAD. Next, based on tertiles, we divided the subjects into three groups. After adjusting for all relevant risk factors, we found that patients with the higher tertile of SHR were associated with an increased risk of multi-vessel CAD compared to those with the lowest tertile. Patients were stratified according to glucose metabolism status into NGR, Pre-DM and DM groups to observe the association between SHR and multi-vessel CAD. Unlike the NGR group, we found SHR was significantly correlated with the higher risk of multi-vessel CAD in the Pre-DM and DM groups, and the higher tertile of SHR was more significantly correlated with the occurrence of multi-vessel CAD than the lowest tertile.

There is a rich literature available suggesting that stress hyperglycemia is transient hyperglycemia secondary to neurohormonal disorders and inflammatory responses [5, 17, 18]. Besides, stress hyperglycemia is very common in critical illness and is a sign of disease severity [19–21]. Roberts et al. first proposed that SHR was not affected by background glycemia and could better evaluate the true blood glucose status of patients [12]. Multi-vessel CAD is associated with a high risk of death and adverse events [3]. In this respect, compared with single-vessel CAD, multi-vessel CAD increased the difficulty of percutaneous coronary intervention and had a worse prognosis [22], and has attracted much interest in recent years. Ample literature suggests that SHR is associated with increased risk of adverse cardiovascular clinical outcomes and in-hospital mortality and is an independent predictor of poor prognosis [5–10, 23].

Chu et al. suggested that high SHR was independently related to large thrombus burden and had a better predictive value for adverse complications and events than admission blood glucose level [24]. Moreover, Kojima et al. found that high SHR was significantly associated with a poorer long-term prognosis in non-diabetic individuals [25]. Cui et al. also demonstrated a strong positive correlation between SHR and long-term mortality in acute myocardial infarction (AMI) patients with or without diabetes [26]. However, Schmitz et al. advocated that SHR was significantly associated with higher short-term mortality in patients with AMI, and was only found to be associated with higher long-term mortality in patients with diabetes [27]. Besides, Chen et al. reported that SHR was an effective predictor of in-hospital outcomes in patients > 75 years of age with AMI, especially in non-diabetic individuals [28]. Last but not least, results of a large observational study based on 274 centers in China by Xu et al., showed that SHR was independently associated with the risk of major adverse cardiovascular events (MACEs) and all-cause mortality in ST-segment elevation myocardial infarction (STEMI) patients [15].

Our study also corroborated the adverse effect of SHR on cardiovascular disease and provided hitherto undocumented evidence that SHR was an independent risk factor for CAD severity; high SHR was closely related to multi-vessel CAD, especially in pre-DM and DM populations. Previous studies suggested that pre-DM was an elevated risk state for cardiovascular events [29–32]. Besides, a meta-analysis of 53 prospective studies showed that pre-DM was associated with an increased risk of CAD [33]. Schlesinger et al. also summarized the results of 95 meta-analyses and found that pre-DM was positively associated with the risk of all-cause mortality and cardiovascular outcomes [34]. These results raise awareness of the need to dynamically monitor blood glucose changes in patients with and without diabetes in the future.

In future clinical practice, we can measure the admission glucose and calculate SHR for all CAD patients, preliminarily assess the severity of condition, and make more timely, accurate and appropriate treatment according to the results, which can also reduce the delay of the condition of critically ill patients with not obvious clinical symptoms. For high-risk groups of CAD, dynamic monitoring of SHR changes can also be used to understand the changes of the condition, so as to seek medical treatment in time and improve the prognosis.

## Strengths and limitations

This is the first study to explore the relationship between SHR and CAD severity, providing the foothold for the prediction and diagnosis of multi-vessel CAD. However, this study still had some limitations. The study population came from a single center, the sample size was small, and information on past medication history was not collected. Accordingly, the generalizability of our results is limited. Further research is needed to validate our findings.

## Conclusion

In summary, our study indicated that high SHR was significantly related to increased risk of multi-vessel CAD, and SHR is a predictor of CAD severity, especially for those with Pre-DM and DM. Risk management of such populations can be done through monitoring the SHR and better health management programs after admission.

## Data Availability

The datasets used and/or analyzed during the current study are available from the corresponding author upon reasonable request.

## Abbreviations

CAD: Coronary artery disease
CHD: Coronary heart disease
CAG: Coronary angiography
SHR: Stress hyperglycemia ratio
BMI: Body mass index
NGR: Normal glucose regulation
Pre-DM: Pre-diabetes mellitus
DM: Diabetes mellitus
HbA1c: Glycated hemoglobin A1c
TC: Total cholesterol
TG: Triglycerides
HDL-C: High-density lipoprotein cholesterol
LDL-C: Low-density lipoprotein cholesterol
UA: Uric acid
BNP: B-type natriuretic peptide
CRP: C-reactive protein
Cr: Creatinine
LAD: Left atrial diameter
LVDd: Left ventricular end diastolic diameter
LVDs: Left ventricular systolic diameter
IVS: Interventricular septal thickness
LVPW: Left posterior wall thickness
LVEF: Left ventricular ejection fraction
OR: Odds ratios
CI: Confidence interval
β: Regression coefficient
ROC: Receiver operating characteristic
AUC: Area under the curve
AMI: Acute myocardial infarction
MACEs: Major adverse cardiovascular events
STEMI: ST-segment elevation myocardial infarction
